# Serum cell-free DNA methylation of *OPCML* and *HOXD9* as a biomarker that may aid in differential diagnosis between cholangiocarcinoma and other biliary diseases

**DOI:** 10.1186/s13148-019-0634-0

**Published:** 2019-03-04

**Authors:** Wiphawan Wasenang, Ponlatham Chaiyarit, Siriporn Proungvitaya, Temduang Limpaiboon

**Affiliations:** 1 Centre for Research and Development of Medical Diagnostic Laboratories, Faculty of Associated Medical Sciences, Khon Kaen University, Khon Kaen, 40002 Thailand; 2 Biomedical Sciences, Graduate School, Khon Kaen University, Khon Kaen, 40002 Thailand; 3 Research Group of Chronic Inflammatory Oral Diseases and Systemic Diseases Associated with Oral Health, Department of Oral Diagnosis, Faculty of Dentistry, Khon Kaen University, Khon Kaen, 40002 Thailand; 4 Cholangiocarcinoma Research Institute, Faculty of Medicine, Khon Kaen University, Khon Kaen, 40002 Thailand

**Keywords:** Cell-free DNA, DNA methylation, Differential biomarker, Misdiagnosis, MS-HRM

## Abstract

**Background:**

Cholangiocarcinoma (CCA) is a fatal cancer of the bile duct epithelial cell lining. The misdiagnosis of CCA and other biliary diseases may occur due to the similarity of clinical manifestations and blood tests resulting in inappropriate or delayed treatment. Thus, an accurate and less-invasive method for differentiating CCA from other biliary diseases is inevitable.

**Methods:**

We quantified methylation of *OPCML*, *HOXA9*, and *HOXD9* in serum cell-free DNA (cfDNA) of CCA patients and other biliary diseases using methylation-sensitive high-resolution melting (MS-HRM). Their potency as differential biomarkers between CCA and other biliary diseases was also evaluated by using receiver operating characteristic (ROC) curves.

**Results:**

The significant difference of methylation levels of *OPCML* and *HOXD9* was observed in serum cfDNA of CCA compared to other biliary diseases. Assessment of serum cfDNA methylation of *OPCML* and *HOXD9* as differential biomarkers of CCA and other biliary diseases showed the area under curve (AUC) of 0.850 (0.759–0.941) for *OPCML* which sensitivity, specificity, positive predictive value (PPV), negative predictive value (NPV), and accuracy were 80.00%, 90.00%, 88.88%, 81.81%, and 85.00%, respectively. The AUC of *HOXD9* was 0.789 (0.686–0.892) with sensitivity, specificity, PPV, NPV, and accuracy of 67.50%, 90.00%, 87.09%, 73.46%, and 78.75%, respectively. The combined marker between *OPCML* and *HOXD9* showed sensitivity, specificity, PPV, and NPV of 62.50%, 100%, 100%, and 72.72%, respectively, which may be helpful to prevent a misdiagnosis between CCA and other biliary diseases.

**Conclusions:**

Our findings suggest the application of serum cfDNA methylation of *OPCML* and *HOXD9* for differential diagnosis of CCA and other biliary diseases due to its less invasiveness and clinically practical method which may benefit the patients by preventing the misdiagnosis of CCA and avoiding unnecessary surgical intervention.

**Electronic supplementary material:**

The online version of this article (10.1186/s13148-019-0634-0) contains supplementary material, which is available to authorized users.

## Background

Cholangiocarcinoma (CCA) is a fatal cancer of the bile duct epithelium. Currently, the incidence and mortality rates of CCA have been increasing worldwide. The incidence of CCA which is as high as 71.3 per 100,000 in males and 31.6 in females has been recorded particularly in the northeastern Thailand where CCA is strongly associated with liver fluke, *Opisthorchis viverrini* infection [1, 2]. The majority of CCA patients are clinical silencing which symptomatic development has been presented at advanced stages leading to poor prognosis and worse clinical outcomes. Surgical resection is the best choice for curative treatment, but most CCA patients are diagnosed with unresectable diseases. The median survival time of CCA with unresectable cases is 6–12 months [3]. Treatment of patients with advanced stages either by chemotherapy or radiotherapy has shown unfavorable effectiveness [4]. The diagnostic procedures of CCA include clinical presentation, blood testing, radiological imaging, and histopathological examination [5]. Although diagnosis of CCA is definitely confirmed by histopathology, this approach is highly invasive for the patients. Moreover, patients with other biliary diseases such as benign biliary tumors, cholecystitis, choledocholithiasis, and cholangitis have clinical presentation, blood chemistry of liver function test and tumor markers of carbohydrate antigen 19–9 (CA19–9) and carcinoembryonic antigen (CEA), and imaging feature of biliary obstruction similarly to CCA, which make it difficult to distinguish CCA from other biliary diseases [6–8]. Some cases of benign biliary diseases diagnosed as CCA are managed with redundant major resections [9–13]. Thus, an accurate differential diagnosis between CCA and other biliary diseases is much essential for effective treatment modality. Several studies have established bile biomarkers for differentiating CCA from other biliary diseases. Budzynska et al. [14] studied bile levels of neutrophil gelatinase-associated lipocalin (NGAL) that can potentially discriminate patients with CCA from benign biliary stenosis. They found that the sensitivity and specificity of NGAL levels were 77.3% and 72.2%, respectively. DNA methylation of *CDO1*, *CNRIP1*, *SEPT9*, and *VIM* in biliary brush samples was evaluated for discriminating CCA from primary sclerosing cholangitis with the sensitivity and specificity of 85% and 98%, respectively [15]. Rose et al [16] determined carcinoembryonic antigen-related cellular adhesion molecule 6 (CEACAM6) in bile samples of CCA and benign biliary diseases, which the sensitivity and specificity were 87.5% and 69.1%, respectively. Nevertheless, bile and biliary brush sampling are an invasive method that may not be practical for all cases. Therefore, serum biomarkers would be a good choice for differentiating CCA from other biliary diseases. Previous studies have shown that serum biomarkers such as CA19–9, CEA, CYFRA 21-1, MMP7, and NGAL could be used to differentiate CCA from other biliary diseases but the sensitivity and specificity were still unsatisfied [17–19].

Recently, investigation of cell-free DNA (cfDNA) has gained more attention for cancer biomarkers. CfDNA, a fragment of DNA, is released into the bloodstream by physiologic and pathologic mechanisms. In cancer patients, cfDNA is mainly derived from apoptotic and necrotic tumor cells which contain genetic abnormalities and epigenetic aberrations such as point mutations, loss of heterozygosity (LOH), microsatellite instability (MSI), and DNA methylation [20]. It has been suggested that cfDNA could be used in clinical practice as a potential biomarker for diagnosis, prognosis, and prediction of cancers because of its minimally invasive technique and less expensive cost for evaluation.

Aberrant DNA methylation has been evidenced as an early event which supports genetic alterations during tumorigenesis and remained existed in advanced stage, in which altered DNA methylation accelerated tumor progression leading to poor clinical outcomes [21]. It has been reported in various human cancers including CCA [22–24]. Our previous study demonstrated that hypermethylation of *OPCML* was highly frequent in CCA tissues but not in normal adjacent (72% vs 0%) [23]. Hypermethylation of *OPCML* was also observed in many cancers such as ovarian cancer [25, 26], non-small-cell lung carcinoma [27], brain tumor [28], bladder cancer [29], and colorectal cancer [30]. Opioid binding protein/cell adhesion mole1cule-like (*OPCML*) gene located on chromosome 11q25 encodes a member of the IgLON subfamily in the immunoglobulin protein superfamily that acts as a glycosylphosphatidylinositol (GPI)-anchored cell adhesion-like molecule expressing in the nervous system especially cerebellum and also in kidney, heart, liver, colon, placenta, pancreas, and testis [26, 28]. OPCML mediates cell-cell adhesion and recognition, and promotes selective neuronal growth and axon migration [28, 31]. Low expression of *OPCML* due to promoter hypermethylation promotes cell proliferation and short survival in gastric cancer [32].

Methylation array data showed that hypermethylation of *HOXA9* and *HOXD9* was observed in 86.3% (88/102) and 89.2% (91/102) of CCA tissue samples, respectively, but not in normal adjacent [33]. The *HOX* (homeobox) family has four *HOX* gene clusters; *HOXA*, *HOXB*, *HOX*C, and *HOXD* which are located on four different chromosomes. They encode homeoproteins which act as critical transcription factors for embryogenesis and differentiation during normal embryonic development [34]. HOXA9 acts as a transcription factor that regulates gene expression involving morphogenesis and differentiation during normal embryonic development. Promoter hypermethylation of *HOXA9* was found in oral [35, 36], breast [37], ovarian [38], bladder [39], and non-small cell lung cancer [40] in which *HOXA9* functions as a tumor suppressor gene. Low expression of *HOXA9* as a result of promoter hypermethylation could promote cell proliferation, invasion, and metastasis in breast cancer [37]. HOXD9 acts as a transcription factor involving vertebral column and forelimb development [41]. *HOXD9* is highly expressed in normal tissues including kidney, testis, colon, spleen, placenta, and bladder but poorly in brain. However, high expression of *HOXD9* transcript and protein was observed in glioma cell lines and brain tumor tissues suggesting the contribution of HOXD9 in cell proliferation and/or survival [42]. Moreover, the study in hepatocellular carcinoma suggested that HOXD9 could act as an oncogene which promotes cell migration, invasion, and metastasis [43]. By contrast, low expression of HOXD9 transcript and protein due to promoter hypermethylation was noticed in melanoma brain metastasis by which DNA methylation of *HOXD9* was significantly higher than that in early stages. Besides, melanoma patients with hypermethylated *HOXD9* in lymph node metastasis showed poorer disease-free and overall survival [44]. Promoter hypermethylation of *HOXD9* was also found in astrocytomas [45]. According to hypermethylation of *OPCML*, *HOXA9*, and *HOXD9* frequently found in CCA tissues, we raised the questions whether this phenomenon could be found in serum cfDNA of CCA and other biliary diseases, and could be used to differentiate CCA from other biliary diseases. Hence, we quantitated serum cfDNA methylation levels of *OPCML*, *HOXA9*, and *HOXD9* in CCA patients and other biliary diseases as well as evaluated their potential as a differential biomarker for CCA.

## Methods

### Serum collection and DNA extraction

Forty serum samples of each group including CCA and other biliary diseases were obligingly supplied by the Cholangiocarcinoma Research Institute, Khon Kaen University, Khon Kaen, Thailand. Patients with suspected CCA subsequently underwent surgical resections which were definitely diagnosed by histopathological examination (Table 1). Written informed consent was obtained from all patients. This study was approved by the Khon Kaen University Ethics Committee for Human Research (HE551066). Serum samples were centrifuged at 16,000×*g* for 3 min and then approximately 1 mL of supernatant was collected and stored at − 80 °C until DNA extraction. Serum cfDNA was extracted by a modified phenol-chloroform technique [46]. In brief, 1 mL of serum was digested by proteinase K solution containing 20 μL of proteinase K (final concentration 20 mg/mL), 100 μL of 250 mM EDTA and 750 mM NaCl, and 100 μL of 10% SDS, then the mixed solution was incubated at 56 °C for 2 h, followed by 25:24:1 phenol:chloroform:isopropyl extraction and ethanol DNA precipitation. CfDNA pellet was dissolved with sterile deionized water in a total volume of 25 μL and stored at − 20 °C until bisulfite modification step.

**Table 1:** Patients enrolled in the studied group

Diagnosis	Number of cases
CCA group	40
Intrahepatic type	27
Perihilar type	13
Other biliary disease group	40
Chronic cholecystitis	19
Papillary adenoma	9
Choledochal cysts	4
Cholangitis	4
Cholelithiasis	2
Choledocholithiasis	1
Hepatolithiasis	1

### Bisulfite modification

Serum cfDNA (20 μL) containing less than 1 μg was treated with bisulfite using EZ DNA Methylation Gold Kit (Zymo Research, Orange, CA) according to the manufacturer’s protocol. A final volume of 20 μL modified cfDNA was obtained and used immediately as a template for methylation-sensitive high-resolution melting (MS-HRM) analysis or stored at − 20 °C no longer than 4 weeks.

### Methylation-sensitive high-resolution melting

The specific primers of CpG islands related to promoters of *OPCML*, *HOXA9*, and *HOXD9* genes were designed following the UCSC genome browser database (December, 2013) (Table 2) and used to amplify bisulfite-modified DNA. To overcome PCR bias phenomenon in which unmethylated alleles are amplified preferentially to methylated, a limited number of CpG dinucleotides (usually one or two) is included in these primer sequences and should be closed to 5′ end as possible [47]. The primers bind preferentially to methylated sequences resulting in an increase of the sensitivity for detection of methylation in minute DNA sources. PCR amplification and HRM were performed on the ABI 7500 Fast Real-Time PCR System version 2.0.6 (Applied Biosystems, Foster City, CA). The reaction mixture was performed in a final volume of 20 μL containing 2 μL or 8 ng of bisulfite-modified cfDNA or DNA standards, 1x PCR buffer (67 mM Tris, pH 8.4, 16.6 mM ammonium sulfate, and 0.1% Tween 20), 2.5 mM MgCl_2_, 200 μM of each dNTP, 300 nM of each primer, 3 μM SYTO9 (Invitrogen, Carlsbad, CA), 0.5 unit of Platinum Taq DNA polymerase (Invitrogen). The optimal conditions of MS-HRM are shown in Table 2. The amplification step was composed of an initial denaturation at 95 °C for 10 min, followed by 40 cycles of denaturation at 95 °C for 15 s and combined annealing and extension in one step as described in Table 2 for 1 min. HRM step consisted of 95 °C for 10 s to denature PCR product, followed by reannealing at 65 °C for 1 min and slowly warmed by continuous acquisition to 95 °C with 1% ramp rate (°C/s). The standard of DNA methylation including 100, 50, 25, 12.5, 6.25, 3.125, 1.56, and 0% was obtained by mixing bisulfite modified fully methylated (100%) and unmethylated DNA sequences. Each reaction was performed in triplicate with no DNA template control included in each experiment. The HRM data were analyzed using High Resolution Melting Software version 2.0.1 (Applied Biosystems). The value of differential fluorescence of each methylation control against 0% methylation was used to generate a standard curve. The linear equation of each MS-HRM was performed in Microsoft Excel 2007 and used for quantification of methylation level of individual genes in clinical samples.

**Table 2:** Primer sequences and optimal MS-HRM conditions of *OPCML*, *HOXA9*, and *HOXD9*

Gene	Primer	Primer sequence 5′- > 3′	UCSC genome browser (December, 2013)	Product (bp)	MgCl_2_ (mM)	*T*_a_ (°C)
*OPCML*	F	CGATCGGGTTGTAGAGGA	chr11: 132943630–132943731	101	2.5	63
	R	CGCATCTAAAACCCCAAAAC				
*HOXA9*	F	AATGCGATTTGGTTGTTTTTTT	chr7: 27165810–27165963	153	2.5	63
	R	CCCCATACACACACTTCTTAAAC				
*HOXD9*	F	GATCGAGGGTTGTAAGAAGAAG	chr2: 176122435–176122541	106	2.5	65
	R	CCCGACCTAACCTAACCC				

*T*_*a*_ annealing temperature

### Statistical analysis

The statistical analysis was performed using SPSS version 16.0 for windows (SPSS Inc., Chicago, IL) and Graph Pad Prism version 6.0 for windows (Graph Pad software, San Diego, CA). Testing for normality of each parameter was determined by Kolmogorov-Smirnov test. The comparison of blood chemistry of liver function test, tumor markers, and methylation levels of *OPCML*, *HOXA9*, and *HOXD9* between CCA and other biliary diseases was determined using either student’s *t* test or Mann-Whitney test. The comparison of DNA methylation of these genes between intrahepatic and extrahepatic CCA was also analyzed using Mann-Whitney test. The assessment of differential biomarker was performed by using receiver operating characteristic (ROC) curve analysis. The cut-off value of each biomarker was determined using Youden index formula: sensitivity + specificity-1, in which the highest sensitivity and specificity were selected. *P* < 0.05 was considered as statistically significant.

## Results

### Blood tests are ineffective for differentiating CCA from other biliary diseases

The enrolled patients were examined for their blood tests including liver function tests (cholesterol, total protein, albumin, globulin, total bilirubin, direct bilirubin, aspartate aminotransferase (AST), alanine aminotransferase (ALT), and alkaline phosphatase (ALP)) and tumor markers (CA19–9 and CEA) before being underwent surgical resection. We found that total bilirubin, direct bilirubin, AST, ALT, ALP, CA19–9, and CEA in both of CCA and other biliary disease group were abnormally elevated. However, no significant difference of liver function tests and tumor markers between CCA and other biliary disease group was observed (*P* > 0.05) (Table 3).

**Table 3:** Comparison of liver function tests and tumor markers between CCA and other biliary diseases

Parameter	Other biliary (mean ± SD)	CCA (mean ± SD)	*P* value
Liver function tests			
Cholesterol	203.1 ± 84.44	194.0 ± 54.10	0.565
Total protein	7.12 ± 0.98	7.41 ± 1.23	0.252
Albumin	3.65 ± 0.65	3.76 ± 0.64	0.443
Globulin	3.48 ± 0.69	3.6 ± 0.91	0.535
Total bilirubin	6.64 ± 10.82	4.16 ± 15.48	0.408
Direct bilirubin	5.24 ± 8.64	2.84 ± 10.17	0.258
ALT	70.15 ± 71.91	60.40 ± 52.13	0.489
AST	99.85 ± 125.2	114.1 ± 226.5	0.729
ALP	300.9 ± 400.2	187.8 ± 149.8	0.098
Tumor markers			
CA19–9	364.3 ± 430.5	415.2 ± 418.8	0.628
CEA	38.26 ± 181.7	53.76 ± 178.9	0.738

### Methylation of *OPCML* and *HOXD9* is a high potential biomarker for differential diagnosis of CCA

**Fig. 1:**
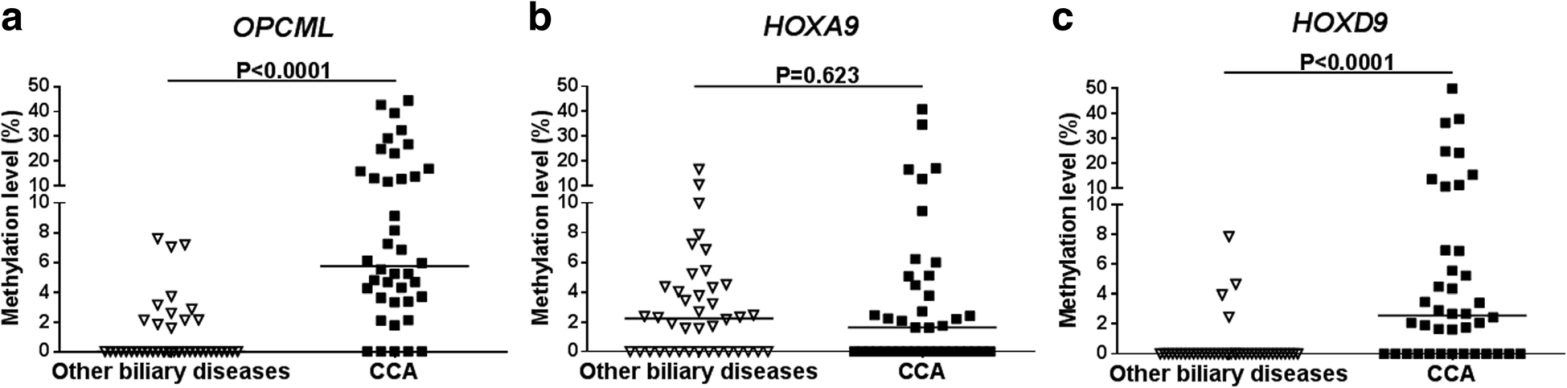
Scatter plots of *OPCML*, *HOXA9*, and *HOXD9* methylation in serum cfDNA between other biliary diseases and CCA patients. **a** *OPCML*, **b** *HOXA9*, and **c** *HOXD9* methylation levels were determined by MS-HRM. The Mann-Whitney *U* test was used to compare between these groups. *P* values < 0.05 were considered statistically significant

**Fig. 2:**
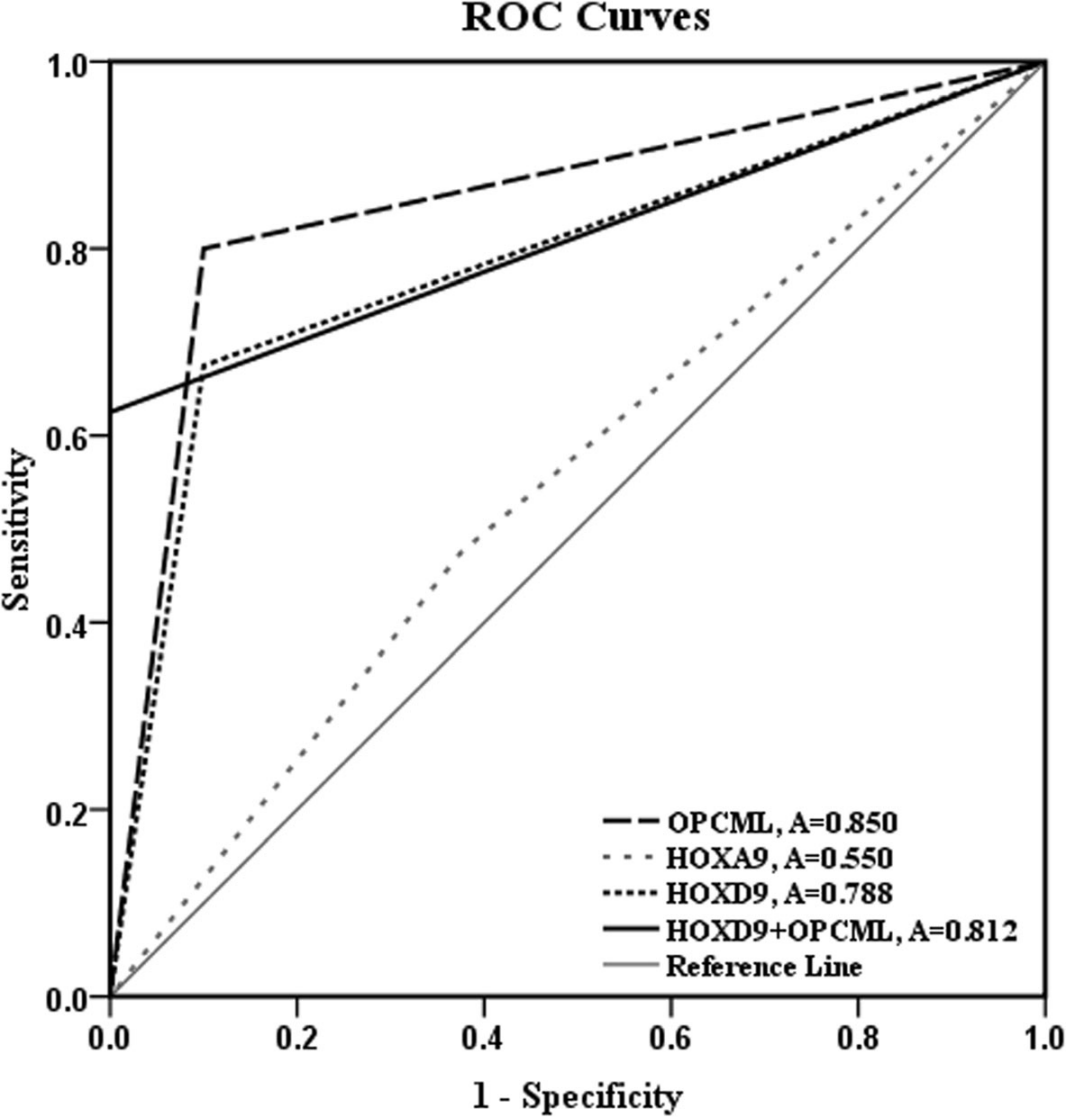
Receiver-operating characteristic (ROC) curves for discriminating CCA patients from other biliary diseases

MS-HRM assay of *OPCML*, *HOXA9*, and *HOXD9* methylation in serum cfDNA was determined by using standard serial dilution including 0, 1.56, 3.125, 6.25, 12.5, 25, 50, and 100% methylation controls (Additional file 1: Figure S1–S3). The lower and upper detection limits of all genes were 1.56% and 50% methylation, respectively. All serum cfDNA derived from CCA and other biliary samples were successfully amplified by which the amplified products were further detected for their methylation levels. The median methylation level of *OPCML*, *HOXA9*, and *HOXD9* in serum cfDNA of CCA was 5.73% (0–44.46%), 1.62% (0–40.97%), and 2.57% (0–50%), respectively, whereas that of other biliary disease group was 0% (0–7.59%), 2.24% (0–16.71%), and 0% (0–7.86%), respectively. The methylation level of *OPCML* and *HOXD9* in serum cfDNA of CCA was significantly higher than that of other biliary group (*P* < 0.0001 and *P* < 0.0001, respectively), while that of *HOXA9* was not significantly different (*P* = 0.623) (Fig. 1). Although the methylation level of *OPCML*, *HOXA9*, and *HOXD9* between intrahepatic and extrahepatic CCA type was not statistically significant difference (*P* > 0.05), there was a trend of high methylation in intrahepatic CCA (Additional file 1: Figure S4). The positive methylated cases in both of CCA and other biliary group were considered based on the lower detection limit (1.56%). The frequency of *OPCML* methylation in serum cfDNA of CCA was 87.5% (35/40), whereas other biliary disease group was 30% (12/40). Moreover, the methylation of *HOXD9* in serum cfDNA of CCA was also frequently detected in 67.5% (27/40) while in other biliary disease group was found only in 10% (4/40). We assessed serum cfDNA methylation of *OPCML*, *HOXA9*, and *HOXD9* as differential biomarkers for CCA from other biliary group using ROC curve (Fig. 2). The cut-off value of methylation level of *OPCML*, *HOXA9*, and *HOXD9* calculated by using Youden index formula was 3.24%, 1.56%, and 1.56%, respectively. Serum methylated *OPCML* marker showed the highest area under curve (AUC) (0.850, 95% CI (0.759–0.941)), which sensitivity, specificity, positive predictive value (PPV), negative predictive value (NPV), and accuracy were 80.00%, 90.00%, 88.88%, 81.81%, and 85.00%, respectively. High AUC (0.789, 95% CI (0.686–0.892)) was found in serum methylated *HOXD9* marker, with sensitivity, specificity, PPV, NPV, and accuracy of 67.50%, 90.00%, 87.09%, 73.46%, and 78.75%, respectively. Nevertheless, serum methylation of *HOXA9* was not potential for differentiating CCA from other biliary diseases (Table 4). Moreover, the combination of these methylation biomarkers was also evaluated for their potency to distinguish CCA from other biliary diseases by using ROC curve. The combined methylated *OPCML* and *HOXD9* showed 100% specificity and 100% PPV with AUC of 0.812 (0.713–0.911) (Table 4).

**Table 4:** Assessment of *OPCML*, *HOXA9*, and *HOXD9* as differential biomarkers between CCA and other biliary diseases

Gene (cut off)	% Sensitivity	% Specificity	% Accuracy	%PPV	%NPV	AUC (95%CI)	*P* value
*OPCML* (3.24%)	80.00 (32/40)	90.00 (36/40)	85.00 (66/80)	88.88 (32/36)	81.81 (36/44)	0.850 (0.759–0.941)	< 0.0001
*HOXD9* (1.56%)	67.50 (27/40)	90.00 (36/40)	78.75 (63/80)	87.09 (27/31)	73.46 (36/49)	0.788 (0.686–0.892)	< 0.0001
*HOXA9* (1.56%)	47.50 (19/40)	62.50 (25/40)	55.00 (44/80)	55.58 (19/34)	54.34 (25/46)	0.550 (0.423–0.676)	0.441
*OPCML* + *HOXD9*	62.50 (25/40)	100.00 (40/40)	81.25 (65/80)	100.00 (25/25)	72.72 (40/55)	0.812 (0.713–0.911)	< 0.0001
*OPCML* + *HOXA9*	30.00 (12/40)	97.50 (39/40)	63.75 (51/80)	92.30 (12/13)	58.20 (39/67)	0.637 (0.515–0.759)	0.034
*HOXA9* + *HOXD9*	25.00 (10/40)	97.50 (39/40)	61.25 (49/80)	90.90 (10/11)	56.52 (39/69)	0.612 (0.488–0.736)	0.083
*OPCML, HOXA9* and *HOXD9* (≥ 2 marker)	72.50 (29/40)	95.00 (38/40)	83.75 (67/80)	93.54 (29/31)	77.55 (38/49)	0.837 (0.743–0.931)	< 0.0001
*OPCML + HOXA9 + HOXD9* (3 markers)	22.5 (9/40)	100 (40/40)	61.25 (49/80)	100 (9/9)	56.34 (40/71)	0.613 (0.488–0.737)	0.083

## Discussion

Most CCA patients usually present at advanced stage when initially diagnosed to have the disease. Although surgical resection is the best curative regimen for CCA patients, resectable cases can be performed only in the early stage. To date, there is no specific method for diagnosis of CCA; however, clinical symptoms, blood chemistry testing, radiological imaging, and histopathological examination have been used in combination for diagnosis of CCA [5]. Unfortunately, the clinical manifestations, liver function tests, and tumor markers of CA19–9 and CEA level of CCA resemble other biliary diseases making it difficult to definitely diagnose. The differential diagnosis between CCA and other biliary diseases was performed based on pathologic evidences that are usually invasive methods and complicated risks, in which safer methods are required. The definite imaging modalities for differential diagnosis of CCA are multidetector computed tomography (MDCT) and magnetic resonance cholangiopancreatography (MRCP) [48]. The sensitivity and specificity of MDCT in differential diagnosis of CCA were 95.8% and 84.6%, respectively [49]. The sensitivity and specificity of MRCP in differential diagnosis between CCA and benign biliary diseases were 95.83% and 100%, respectively [50]. However, the diagnostic accuracy of MRCP was dependent on the location of the biliary stricture and limited image quality [50, 51]. Although MDCT and MRCP are non-invasive methods, they remain expensive and not readily available in all areas for investigating CCA**.** Our present study indicated that the cut-off value of methylation level of *OPCML* and *HOXD9* is the denominator which can be used for distinguishing CCA from other biliary diseases. The percentage of methylation of *OPCML* and *HOXD9* in other biliary diseases is lower than that in CCA. Notably, the high percentage of methylation of these genes may reflect gene silencing and promote cholangiocarcinogenesis. Thus, approach of serum biomarkers may aid in differential diagnosis between CCA and other biliary diseases at the beginning before sophisticated investigation. Presently, there are serum biomarkers that are clinically used as diagnostic and prognostic markers for CCA such as CA19–9 and CEA. However, their sensitivity and specificity remain unsatisfied. Our study indicated the failure of CA19–9 and CEA in distinguishing CCA from other biliary diseases. Recently, there are a few studies searching for differential serum biomarker of CCA. The study of Janvilisri et al. [52] which identified proteins in serum of CCA and benign biliary tract diseases using proteomic approach showed the top five candidate proteins; FAM19A5, MAGED4B, KIAA0321, RBAK, and UPF3B, which may potentially distinguish CCA from benign biliary tract diseases. However, these proteins have not been tested for their sensitivity and specificity. Liu et al. [53] determined serum level of transthyretin in CCA and benign hepatobiliary diseases with sensitivity of 76.8% and specificity of 93.8%.

There are many techniques for the detection of methylation such as methylation-specific PCR (MSP), pyrosequencing, bisulfite sequencing, and MS-HRM. MSP technique is a widely used technique for screening methylation. However, there are two primer sets amplifying either methylated or unmethylated sequences in the procedure that make it difficult to control equal PCR efficiency. Moreover, it is laborious and time consuming which post-PCR processing is required [54]. Thus, MSP is not suitable for detection of methylation in cfDNA due to its low sensitivity. Quantitative MSP (QMSP) has been established to overcome these pitfalls by using real-time PCR machine. This technique is simple, highly specific, cost-effective, and less time-consuming. The limitation of QMSP is the amplification will occur only when all CpG sites are methylated leading to less sensitivity which is not suitable for detecting a small number of methylated sequences in minute DNA sources containing high unmethylated background [55]. Pyrosequencing and bisulfite sequencing techniques are able to detect methylation level at individual CpG sites which QMSP and MS-HRM cannot do. However, pyrosequencing requires several enzymes for real-time DNA synthesis in the sequencing reaction. The stability, fidelity, specificity, and sensitivity of the test are necessary for the optimal performance of the enzymes used in the reaction [56]. Although bisulfite sequencing is a gold standard method for methylation detection, it is less sensitive, labor-intensive, and time-consuming which it is unsuitable for clinical setting [57]. MS-HRM is a technique for detection of overall methylation percentage in the entire amplicon. The difference in base composition between methylated and unmethylated DNA after bisulfite modification results in different melting temperatures. Even though MS-HRM is appropriate for rapid screening of overall methylation status, the number of CpG sites and size of the amplicon are important for generating percentage of methylation. Nevertheless, this technique is simple, cost-effective, highly sensitive and reproducible, and less time-consuming which can be performed in a real-time PCR machine. There are some reports applying MS-HRM for methylation detection in clinical samples. For example, the detection of serum DNA methylation in intrauterine growth retardation infants [58], in pregnant women [59], and in nasopharyngeal carcinoma patients [60]. Moreover, HRM is suitable in many clinical applications such as point mutation detection, single-nucleotide polymorphism (SNP), and microbial genotyping [61].

Circulating cfDNA is released into blood circulation from different sources including the primary tumor, circulating tumor cells, metastatic tumor, and normal cell types such as hematopoietic and stromal cells [62]. During tumor development and progression, the increased release of cfDNA in the blood occurs by apoptotic and necrotic cell death. As circulating cfDNA may reflect the characteristics of the primary tumor and even of metastatic tumor in real time, it may be an excellent biomarker for cancer patients [20]. Evaluation of *OPCML* methylation as a diagnostic biomarker in clinical tissue samples has been performed in ovarian cancer [63], cholangiocarcinoma [64], prostate cancer [65], and lung cancer [27]. With the advent of liquid biopsy, it becomes superior to tissue biopsy because of its less invasiveness and clinically practical method. Consequently, *OPCML* methylation in serum cfDNA has been assessed for its value as a diagnostic biomarker in ovarian cancer [66, 67]. There is no evaluation of *OPCML* methylation as a differential marker for cancer patients. Our study showed that serum *OPCML* methylation can be used as a differential biomarker for CCA with high sensitivity and specificity. Although high methylation levels of *OPCML* and *HOXD9* found in sera of CCA patients may indicate tumor progression, no significant differences in tumor size, stage, and survival time were observed between low and high methylation group (Additional file 1: Table S1). The high methylation level of *OPCML* observed in sera of some cases in other biliary diseases such as chronic cholecystitis (7/19) and papillary adenoma (5/9) may reflect an increase risk in developing CCA in the future in which clinical follow up of these patients should be concerned. Previous study in melanoma patients with lymph node metastasis showed the association of hypermethylated *HOXD9* with poor prognosis [44]. However, serum cfDNA methylation of *HOXD9* in human cancer and its application as a tumor biomarker has not been reported. Our present study showed that serum cfDNA methylation of *OPCML* and *HOXD9* could potentially differentiate CCA from other biliary diseases. Interestingly, the combined *OPCML* and *HOXD9* methylation increased the specificity and PPV of the test to 100%, potentially supporting differential diagnosis between CCA and other biliary diseases.

OPCML is a member of a family of GPI-anchored cell adhesion molecules which is localized in plasma membrane. It plays an important role in cell-cell adhesion and negatively regulates specific receptor tyrosine kinases (RTK) by interacting with their extracellular domains which promote proteasomal degradation leading to inhibition of RTK signaling pathway [68]. Previous study in ovarian and breast cancers showed that OPCML can disrupt EGFR-HER2 heterodimerization by binding to HER2 and inhibiting downstream pathway. They also showed that restoration of OPCML expression can sensitize HER2-expressing breast and ovarian cancer cells to both lapatinib and erlotinib. Moreover, high OPCML expression was associated with longer survival in patients with HER2-positive ovarian cancer, and with better response to lapatinib treatment in breast cancer patients [69]. The downregulation of *OPCML* in colorectal and gastric cancers was significantly associated with promoter methylation at a region from − 125 to + 4 bp of the transcription start site (TSS) [30, 32]. We analyzed DNA methylation of *OPCML* at the same area from − 60 to + 41 bp of the TSS which contains ten overlapped CpG sites with the previous studies implicating that promoter DNA methylation of *OPCML* in our study may affect gene silencing in CCA. Low expression of OPCML due to DNA methylation was reported in CCA [23]. Collectively, cfDNA methylation of *OPCML* may potentially be used for monitoring tumor recurrence and response to treatment in CCA. A lot of independent validation would be needed for this to be confirmed. HOXD9 acts as a transcription factor involving in cell morphogenesis which is expressed in various normal epithelial tissues [42]. Promoter DNA methylation of *HOXD9* at a region from − 753 to + 193 bp of the TSS decreased its expression in melanoma. Our study showed ten overlapped CpG sites with the previous study at a region from − 285 to − 179 bp of the TSS [44]. Thus, promoter methylation of *HOXD9* may regulate gene repression in CCA. Loss of HOXD9 may promote dedifferentiation of cholangiocyte leading to CCA development. We are the first to report the use of serum cfDNA methylation as a differential biomarker between CCA and other biliary diseases indicating its applicability for supporting the accurate diagnosis of CCA. However, most of CCA patients enrolled in the present study were advanced stages. Further study in early stage CCA should be warranted for the application of serum cfDNA methylation of *OPCML* and *HOXD9* as an early differential biomarker in CCA.

## Conclusions

In conclusion, detection of serum cfDNA methylation of *OPCML* and *HOXD9* which served as a differential biomarker could be clinically helpful to prevent misdiagnosis between CCA and other biliary diseases due to its simplicity, less invasiveness, and clinically practical method. However, detection of these markers in a large-scale samples as well as more early stage cases included should be warranted before implementation as differential biomarkers for CCA.

## Additional file

Additional file 1: **Figure S1.** The optimization of *OPCML* MS-HRM assay using standard serial dilution series (0–100% methylation). **Figure S2.** The optimization of *HOXA9* MS-HRM assay using standard serial dilution series (0–100% methylation). **Figure S3.** The optimization of *HOXD9* MS-HRM assay using standard serial dilution series (0–100% methylation). **Figure S4.** Scatter plots of *OPCML, HOXA9* and *HOXD9* methylation in serum cfDNA between intrahepatic and extrahepatic CCA patients. **Table S1.** The association of *OPCML* and *HOXD9* methylation with clinicopathological data. (PDF 1317 kb)

## Abbreviations

AUC: Area under curve
CCA: Cholangiocarcinoma
Cf: Cell-free DNA
MS-HRM: Methylation-sensitive high-resolution melting
MSP: Methylation-specific PCR
NPV: Negative predictive value
PPV: Positive predictive value
ROC: Receiver-operating characteristic
RTK: Receptor tyrosine kinases
SNP: Single-nucleotide polymorphism
TSS: Transcription start site
