# Supplementary material for: Serum cell-free DNA methylation of OPCML and HOXD9 as a biomarker that may aid in differential diagnosis between cholangiocarcinoma and other biliary diseases
Source: Clin Epigenetics. 2019 Mar 4;11:39. doi: 10.1186/s13148-019-0634-0 (PMC6399934; doi:10.1186/s13148-019-0634-0)
Supplement: Supplementary file 1 — Figure S1. The optimization of OPCML MS-HRM assay using standard serial dilution series (0–100% methylation). Figure S2. The optimization of HOXA9 MS-HRM assay using standard serial dilution series (0–100% methylation). Figure S3. The optimization of HOXD9 MS-HRM assay using standard serial dilution series (0–100% methylation). Figure S4. Scatter plots of OPCML, HOXA9 and HOXD9 methylation in serum cfDNA between intrahepatic and extrahepatic CCA patients. Table S1. The association of OPCML and HOXD9 methylation with clinicopathological data. (PDF 1317 kb) [file 13148_2019_634_MOESM1_ESM.pdf]

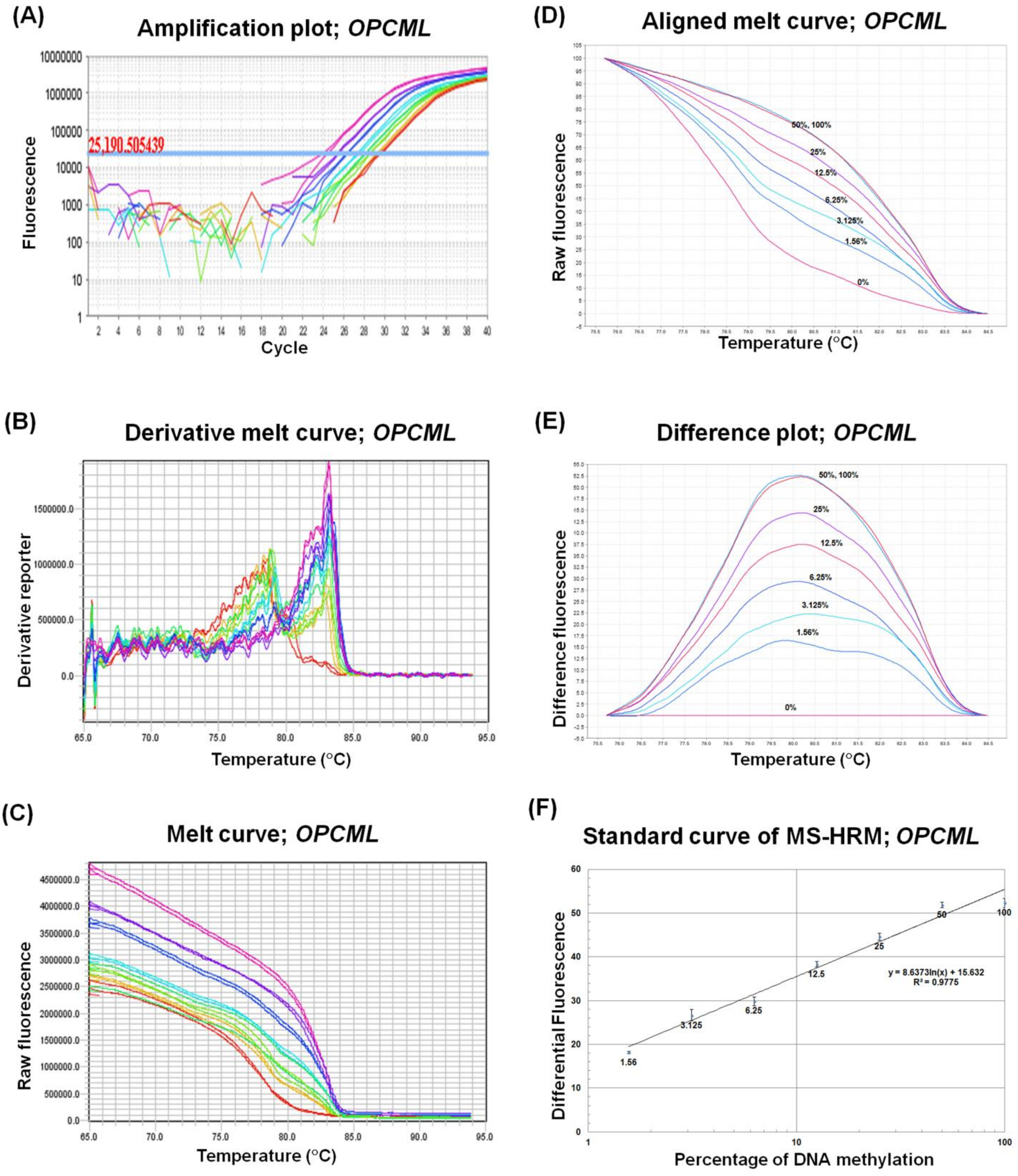

Figure S1. The optimization of *OPCML* MS-HRM assay using standard serial dilution series (0-100% methylation). (A) The amplification plots of serial dilution with equal amount of starting modified DNA demonstrating that modified DNA templates were preferentially amplified in higher methylation controls (B) Derivative melt curve showed two specific peaks, unmethylated (lower melting temperature (Tm)) and methylated alleles (higher Tm). (C) Raw melt curve of HRM. (D) Aligned melt curve plots, the normalized melt curves with different methylation (0-100%) over temperature. (E) The difference plots, the aligned data of each sample against unmethylated control. (F) The linear standard curve of *OPCML* MS-HRM was established by plotting the value of differential fluorescence peak of each control against 0% methylation control. Methylation level of unknown samples was calculated from the linear equation.

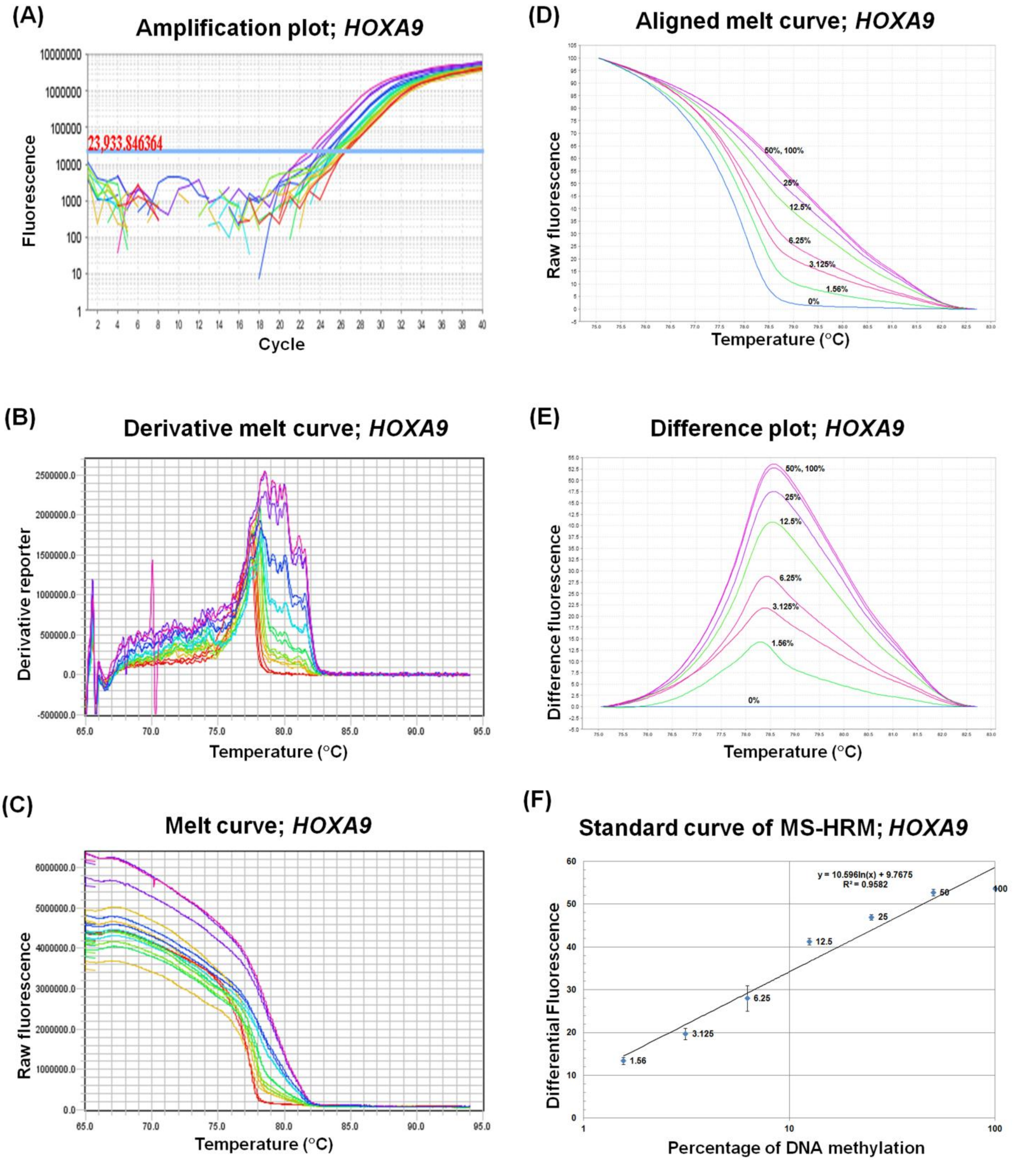

Figure S2. The optimization of *HOXA9* MS-HRM assay using standard serial dilution series (0-100% methylation). (A) The amplification plots of serial dilution with equal amount of starting modified DNA demonstrating that modified DNA templates were preferentially amplified in higher methylation controls (B) Derivative melt curve showed two specific peaks, unmethylated (lower Tm) and methylated alleles (higher Tm). (C) Raw melt curve of HRM. (D) Aligned melt curve plots, the normalized melt curves with different methylation (0-100%) over temperature. (E) The difference plots, the aligned data of each sample against unmethylated control. (F) The linear standard curve of *HOXA9* MS-HRM was established by plotting the value of differential fluorescence peak of each control against 0% methylation control. Methylation level of unknown samples was calculated from the linear equation.

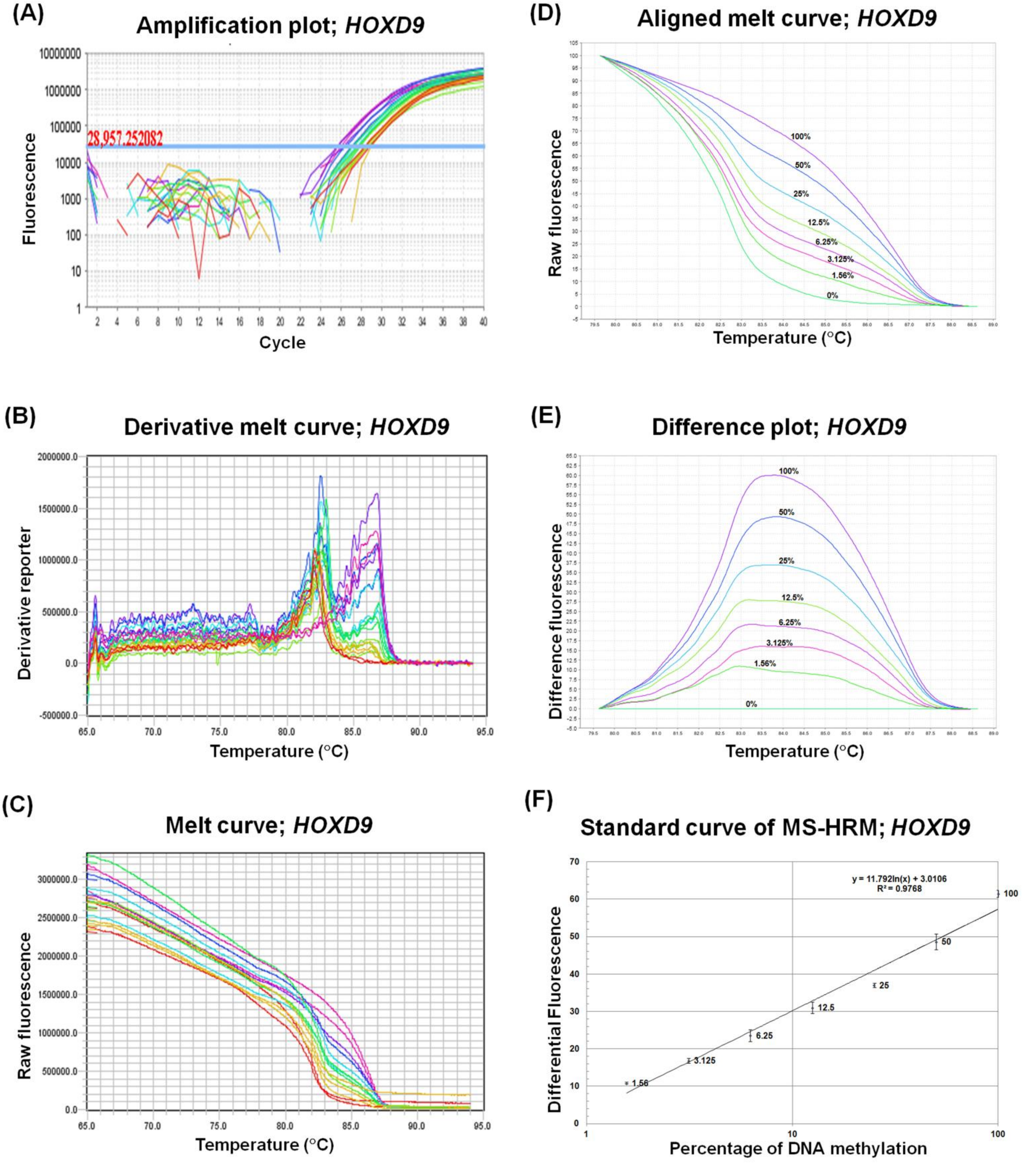

Figure S3. The optimization of *HOXD9* MS-HRM assay using standard serial dilution series (0-100% methylation). (A) The amplification plots of serial dilution with equal amount of starting modified DNA demonstrating that modified DNA templates were preferentially amplified in higher methylation controls (B) Derivative melt curve showed two specific peaks, unmethylated (lower Tm) and methylated alleles (higher Tm). (C) Raw melt curve of HRM. (D) Aligned melt curve plots, the normalized melt curves with different methylation (0-100%) over temperature. (E) The difference plots, the aligned data of each sample against unmethylated control. (F) The linear standard curve of *HOXD9* MS-HRM was established by plotting the value of differential fluorescence peak of each control against 0% methylation control. Methylation level of unknown samples was calculated from the linear equation.

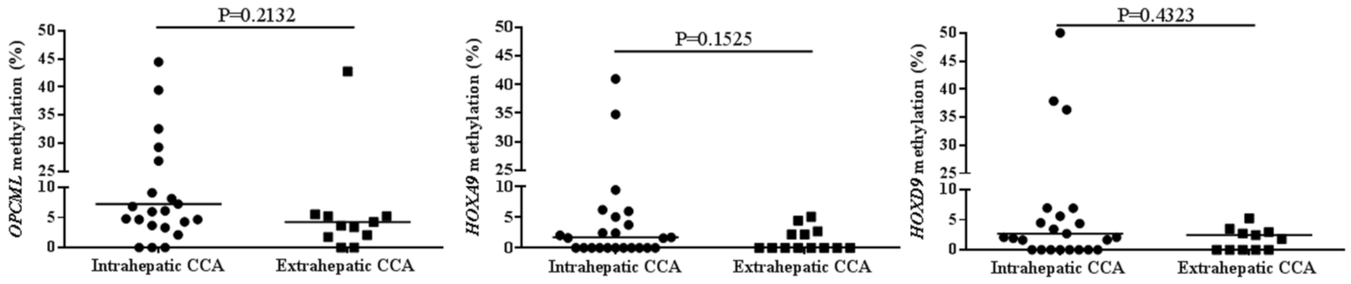

Figure S4. Scatter plots of *OPCML*, *HOXA9* and *HOXD9* methylation in serum cfDNA between intrahepatic and extrahepatic CCA patients. *OPCML*, *HOXA9* and *HOXD9* methylation levels were determined by MS-HRM. The Mann-Whitney U-test was used to compare between these groups. P-values < 0.05 were considered statistically significant.

Table S1. The association of *OPCML* and *HOXD9* methylation with clinicopathological data

Parameters	n (cases)	DNA methylation in CCA serum					
		HOXD9			*OPCML*		
		Negative n (%)	Positive n (%)	P-value	Negative n (%)	Positive n (%)	P-value
Total cases	40						
Age							
≤ 59 years	20	6 (30)	14 (70)	0.333	4 (20)	17 (80)	0.716
>59 years	20	10 (50)	10 (50)		6 (30)	14 (70)	
Gender							
Male	25	9 (36)	16 (64)	0.527	4 (16)	21 (84)	0.135
Female	15	7 (46.7)	8 (53.3)		6 (40)	9 (60)	
Tumor size							
≤ 7 cm	22	9 (40.9)	13 (59.1)	1.000	4 (18.2)	18 (81.8)	0.300
> 7cm	18	7 (38.9)	11 (61.1)		6 (33.3)	12 (66.7)	
Histological grade							
Well differentiated	13	4 (30.8)	9 (69.2)	0.502	4 (30.8)	9 (69.2)	0.540
Moderately differentiated	18	9 (50)	9 (50)		5 (27.8)	13 (72.2)	
Poorly differentiated	9	3 (33.3)	6 (66.7)		1 (11.1)	8 (88.9)	
Stage							
Early (0, I, II)	7	4 (57.1)	3 (42.9)	0.407	2 (28.6)	5 (71.4)	1.000
Late (III, IV)	33	12 (36.4)	21 (63.6)		8 (24.2)	25 (75.8)	
Lymph node metastasis							
Negative	14	5 (35.7)	9 (64.3)	1.000	2 (14.3)	12 (85.7)	0.672
Positive	20	7 (35)	13 (65)		5 (25)	15 (75)	
Median survival time (days)	40	233 (34-1597)	247 (61-1354)	0.876*	248 (34-903)	206.5 (54-1597)	0.855*

*Log rank test
